# Understanding Values in a Large Health Care Organization through Work-Life Narratives of High-Performing Employees

**DOI:** 10.5041/RMMJ.10062

**Published:** 2011-10-31

**Authors:** Orit Karnieli-Miller, Amanda C. Taylor, Thomas S. Inui, Steven S. Ivy, Richard M. Frankel

**Affiliations:** 1 Department of Community Mental Health, University of Haifa, Israel, and Indiana University School of Medicine, Indianapolis, Indiana, USA;; 2 Department of Psychology, Edward Hines Jr. VA Hospital, Hines, IL, USA;; 3 Regenstrief Institute and Indiana University School of Medicine, Indianapolis, Indiana, USA;; 4 Indiana University Health, Indianapolis, Indiana, USA;; 5 Regenstrief Institute, Roudebush VAMC, Indiana University School of Medicine, Indianapolis, Indiana, USA

**Keywords:** Values, work-life narratives, appreciative inquiry, narratives

## Abstract

**Objective—:**

To understand high-performing frontline employees’ values as reflected in their narratives of day-to-day interactions in a large health care organization.

**Methods—:**

A total of 150 employees representing various roles within the organization were interviewed and asked to share work-life narratives (WLNs) about value-affirming situations (i.e. situations in which they believed their actions to be fully aligned with their values) and value-challenging situations (i.e. when their actions or the actions of others were not consistent with their values), using methods based on appreciative inquiry.

**Results—:**

The analysis revealed 10 broad values. Most of the value-affirming WLNs were about the story-teller and team providing care for the patient/family. Half of the value-challenging WLNs were about the story-teller or a patient and barriers created by the organization, supervisor, or physician. Almost half of these focused on “treating others with disrespect/respect”. Only 15% of the value-challenging WLNs contained a resolution reached by the participants, often leaving them describing unresolved and frequently negative feelings.

**Conclusions—:**

Appreciative inquiry and thematic analysis methods were found to be an effective tool for understanding the important and sometimes competing role personal and institutional values play in day-to-day work. There is remarkable potential in using WLNs as a way to surface and reinforce shared values and, perhaps more importantly, respectfully to identify and discuss conflicting personal and professional values.

## INTRODUCTION

Organizational climate and work satisfaction have been studied using a large number of well validated instruments (e.g. Benchmarking Press Ganey: http://www.data-advantage.com/; http://www.pressganey.com/ourSolutions.aspx; Picker: http://www.nrcpicker.com/products-solutions/). These surveys often tap into the attitudes of workers and their levels of satisfaction with work and the organizational environment. The value of climate and work satisfaction tools is the ability to sample large numbers of employees over time and to be able to “benchmark” results against other organizations. The major limitation of these tools is that they often use forced choice questions that dichotomize workplace satisfaction and do not address the complexity of decisions, work processes, social interactions, and actions within the organization.^1^,^2^

Understanding attitudes and behaviors in organizations as done in these surveys is important; however, many of these surveys overlook the values guiding and underlying these individuals’ behavior and attitudes. Values play a significant role in day-to-day work. They give meaning to life and contribute to one’s sense of identity,^3^,^4^ mediate ethical decisions in practice, and guide interactions with patients, colleagues, other professionals, and the public.^5^ Individuals’ values determine which types of situations in the work setting will be experienced as stressful (i.e. value-challenging) and which will bring delight (i.e. value-affirming). The goal of the present study was to learn which values guide high-performing employees’ behaviors, how these values play a role in their day-to-day actions and experiences, and how they influence their work.

To answer these questions, we conducted a study based on work-life narratives (WLNs) of 150 high-performing frontline employees in a large health care organization. In this paper various organization employees and managers were interviewed, except physicians who were the focus of the follow-up study. We sought to understand the values in play when the organization was at its best and when it was most challenged. Narratives afford the teller and the analyst an opportunity to witness and re-live the private professional human engagements that usually remain invisible and unknowable;^6^ they help in describing the context, culture, and complexity of organizations^7^–^10^ and open a “window” into the day-to-day lived experiences and manners in which professionals make decisions.^11^ Narratives embody the story-teller’s values,^12^ and their analysis allows the researcher to understand real situations^13^ and uncover stories that would otherwise remain below the surface.^14^

## STUDY QUESTIONS

In collaboration with a senior vice-president in the organization (author S.S.I.), we identified the following research questions:

- What values do high-performing frontline employees in this organization embody when things go well in their day-to-day work (value-affirming)?
- What values do high-performing frontline employees in this organization embody when their values are challenged (value-challenging)?
- What are the characteristics of the challenging situations?
- How are they managed/resolved?

## METHODS

This was a qualitative study based on the WLNs elicited during 150 face-to-face semistructured interviews lasting 30–45 minutes. The developmental process was based on appreciative inquiry (AI), an organizational change strategy that focuses on what organizations do well and asks how to get more out of what works, rather than fixing what is broken.^15^ Given the focus on what is positive in this organization, the research team decided to interview high-performing employees, in this case defined as having been recognized for their contributions through awards or community consensus. The study was approved by the hospital’s Board of Directors Committee on Values, Ethics, Social Responsibility and Pastoral Services. Twenty employees from the organization volunteered and were trained to conduct the interviews. The interviewers were: 16 chaplains, 3 program directors, and 1 social worker. All were trained in AI methods during a single 3-hour session.

### Interview Guide

To avoid inadvertently biasing the responses, the interviewers were given a scripted interview guide and instructed to follow it as written. The interview guide included: personal meaning and commitment, an appreciative value-affirming WLN about a time/situation/occasion when they and the organization were at their best, and a time when they felt their values were challenged. All interviews were digitally recorded. The recordings were transcribed verbatim and checked by the research team for accuracy.

### Sampling

Snowball sampling was used to select high-performing frontline staff from three of the hospitals which comprise the academic health center. A list of “outstanding” employees, who had been recognized by the organization for their achievements, was made available to aid in the initial selection. In addition, each interviewer asked participants for the names of two or three other employees whose work they considered exemplary and whom they perceived as truly living the organization’s values.^16^ Those recommended were interviewed and the sample “snowballed” until the target of 150 was reached. This number was chosen before the analysis, as our best guess to what would allow us to reach theoretical saturation. This is a large number for a qualitative study to allow various participants from various roles in the hospital to participate and to allow identifying trends in value-affirming versus value-challenging stories.

### Participants and Organization Background

Participants included a diverse sample of hospital employees with varying years of service to the organization (Table 1 and Table 2).

**Table 1:** Participants’ job titles.

**Nurses**	**Managers**	**Educators/Human Resources/Dieticians**	**Therapists/Counselors**	**Office Assistants**	**Service Personnel**	**Did Not State**
50	15	2/5/3	15	15	28	17

**Table 2:** Participants’ length of service in the organization.

**<2 Y**	**2–5 Y**	**6–9 Y**	**>10 Y**	**Did Not State**
22	15	20	66	27

The organization in which these high-performing employees work is a not-for-profit, non-sectarian, health care system. The community sponsors of this system are Indiana University and the Indiana Conference of the United Methodist Church. The Academic Health Center employs more than 10,000 persons and admits 60,000 patients per year. It provides over 1 million out-patient visits annually. The mission of Indiana University Health is to improve the health of the patients and community through innovation and excellence in care, education, research, and service.

## ANALYSIS

We analyzed the WLNs using an immersion/ crystallization method (thematic narrative analysis framework).^17^ The analysis proceeded in several steps:^18^ first, three coders randomly selected the same three employee WLNs, independently highlighting and giving provisional names to sections of transcripts believed to contain value statements. This was done using a technique called a “horizontal pass”, which consisted of reading and re-reading the narratives in their entirety and searching for themes.^19^ Next the coders met, compared and contrasted their findings, and came to consensus on types and levels of themes. Another set of three same interviews was randomly selected, independently coded, and discussed using the results of the last consensus-building round. This process was repeated until agreement was reached on coding content and themes within the WLNs (i.e. trustworthiness). As a trustworthiness check, another member of the research team (T.S.I.) conducted a confirmability audit by separately coding 10% of the interviews and then comparing his findings with those of the other three coders. Once consensus had been achieved among all four coders, a value-coding matrix was developed by clustering provisional categories under larger themes, at which point the remaining narratives were coded (for details see Taylor et al.^18^). During this process one coder (O.K.M.) stepped back from the data to see if some of them could be clustered together. This process included eliminating repetitive value themes (e.g. caring and care for others), dividing the remaining into eight conceptual groups, and recording them in the codebook. Following this the whole group met to refine the coding and added two more conceptual categories. This process resulted in a more fine-grained set of categories (e.g. unselfishness/ self, caring/care for others, and diligence) subsumed under larger conceptual themes (e.g. valuing patients’ well-being).

Consistent with the narrative analysis framework, special attention was paid to different narrative elements in order to understand the meanings embedded in each.^20^,^21^ These elements were derived by asking: 1) What was the core value mentioned in each WLN? (Since many WLNs included multiple values a coding decision was made to include only the most salient value in each WLN.) 2) Who were the main characters (e.g. self, patient, organization)? 3) How were the main characters positioned (i.e. employees connected to patient or organization)?

## RESULTS

The WLNs were filled with complex and often competing values. Analysis of the value-affirming and value-challenging WLNs was conducted separately to identify specific values that were significant in each type. We illustrate the main values with representative examples.

The final coding included 10 broad values, each containing between 1 and 16 subcategories (Table 3).

**Table 3:** Themes and categories identified in appreciative and challenging narratives.

**Themes**	**Categories**
**Valuing patients’ well-being**	Unselfishness/Self Caring/Care for others Generosity Diligence Self-awareness Facilitating relationships Dedication
**Going above and beyond**	Enthusiasm Excellence Social intelligence Trying to understand patients’ and families’ perspectives Adaptability/Flexibility Creativity Being an advocate Get it done Being of service
**Treating others with respect**	Equality Acceptance Fairness Justice Honesty/Integrity Trustworthiness Responsibility Reliability/Dependability
**Feeling part of an organization or team**	Leadership Citizenship Loyalty Teamwork Teaching/Educating Compromise Honor
**Helping and healing**	Humanism Acceptance and acknowledgment of death and dying Hospitality Helpfulness Golden rule Connecting with others Personal experience Hospitality Presence Creating intimacy Holistic care Perspective Non-verbal communication Esthetics Commitment Using my own talents and knowledge
**Expressing passion and emotion**	Happiness/Joy Love: valuing close relations with others Sympathy Hope: optimism, future-mindedness, future orientation Emotional investment
Growing and developing	Mastering new skills, topics, and bodies of knowledge Growth and development Expertise Love of learning
Believing in a higher power	Faith Spirituality – purpose Religiosity
Doing the right thing	Doing the right thing – even if it means risking your job
Gratitude and appreciation	Gratitude and appreciation – being aware of and thankful of the good things that happen Acknowledging work well done

### Value-Affirming WLN Analysis

The main characteristics that were mentioned in the 169 value-affirming WLNs involved the narrator (i.e. self) and the patient and/or patient’s family (80 WLNs), or self and/or patient with the team (61 WLNs), followed by self and the organization (21 WLNs). The remaining seven WLNs were about the self/family or team and the supervisor or physician. The core values most frequently mentioned were:

- Going above and beyond (being of service, creativity and flexibility, getting things done, and advocating for patients) (24.2%)
- Valuing patients’ well-being (caring, generosity, and facilitating relationships) (20.7%)
- Helping and healing (creating a connection with others and helpfulness) (20.1%)
- Feeling part of the organization and team (belonging, teamwork, and teaching others) (17.2%)
- All others (gratitude and appreciation, believing in a higher power, expressing passion emotion, doing the right thing, treating others with respect, and growing and developing) (17.8%)

#### Going Above and Beyond

Going above and beyond focused on personal commitment and enthusiasm, with passionate interest and eagerness to do things to help the patient or family, more than expected. This included sensitivity and awareness of others’ motives and feelings, focusing and understanding the other person’s point of view, and being of service. Within this theme employees were creative in finding ways to help patients. They showed originality and effort in thinking of novel and productive ways to conceptualize and do things (e.g. turning bed, buying hats, bringing dog) to help, as illustrated below:

“We were trying to get one patient to get up and walk in the halls so that he wouldn’t get weak cause he was going to be going home soon. He was just like, ‘is there something to do? You know, I like to walk on the beach, but, just walking in the hall is kind of boring’. So the nurses and I actually brought in a little mat, we put some sand down, we put like a little beach chair and had like a little poster of like a beach scene and some water and finally got him out to kinda walk over there and at least sit and pretend like he was on the beach for a while. We all had fun doing it.”

These employees combined fun, caring, and creativity in their efforts to help a patient, out of concern for his physical and emotional health. Going above and beyond narratives were focused on doing things that are more than expected in the job requirements to help the patients or their family members. This included mediating between patients and other institutions (e.g. financial assistance), being creative in finding ways to assist the individual or to bring joy to patients’ and families’ hospitalization experience.

#### Valuing Patients’ Well-Being

Valuing patients’ well-being included an attitude or way of behaving marked by unselfish concern for the needs and welfare of others. These stories were about being compassionate and/or showing concern. Stories included employees being generous, willing to give money, help, or time freely (magnanimity), as well as being diligent, by working hard and investing effort in doing something for patients’ well-being. Valuing patients’ well-being is focused on a holistic look at people’s needs rather than focused on the medical need, as illustrated in the following WLN:

“We had a two-and-a-half-year-old patient who had been here his whole life … He had to be in isolation, due to an infection. I would go in there every night before I left and rock him to sleep, because his mom was a single mom; she couldn’t be here a lot and I couldn’t stand the thought of him always going to sleep by himself … And there was this Sunday night before he passed away, ... and work called and said, ‘You know he’s not doing real well, we’re really, really busy, I think he needs to be rocked to sleep, and we were wondering if you would come in’. And I said, ‘Absolutely’. So I got in my car and came … he was just sitting there awake. He grabbed the bars and just kind of looked outside and so I went in there and rocked him to sleep.”

When employees described caring for patients they mentioned such issues as: willingness to sacrifice their own comfort to provide the highest-quality care (e.g. coming in on their day off), working late hours, assisting in other units without their own team, and performing services not included in their professional job description (e.g. rocking the child to sleep).

#### Helping and Healing

Many of the value-affirming WLNs described finding ways to help make a patient’s stay in the hospital as comfortable and pleasant as possible. Employees helped fulfill patients’ dying wishes and adjust and cope with their new health status. Employees were able to relate to patient and family needs, even if they fell outside of their defined professional roles or outside of organizational regulations. They listened and addressed personal preferences and religious beliefs. For instance:

“A little boy fell off a lawnmower and his arm had been cut off … this was a very nasty complete amputation. We had the limb in a cooler and the surgeon took a look at it and said to the father: ‘I can’t put that back on because this kid will be frustrated with it and he will be better off with a prosthesis …’ As they were leaving, the father picked up the cooler and I said: ‘You can just leave that here’, and he said: ‘No, I’m taking that’, and I said: ‘Why don’t you let me take care of it and I’ll clean up the cooler and bring it to you?’ He said: ‘No, I’m taking it’, and I said: ‘Could you tell me what you’re going to do with it?’ And he said: ‘Those are the five little fingers that I kissed and wrapped around my fingers and I’m not going to let you throw them away’. Another nurse and I said simultaneously: ‘What cooler?’ I said that we have some things to do over here and you just go out in the hall and we’ll have someone take you to surgery. I think even if I had known that I would have got fired (for doing that), it wouldn’t mean anything to me.”

As these nurses understood the value of the son’s limb for the father, they decided to do what they believed to be the right thing, even though it meant risking their jobs. This is an example of nurses putting patients’ and family members’ wishes above standard hospital policy that mandates that any tissue taken from patients has to be retained and given to pathology, and employing mindful value-based action. It also illustrates the complexity of human and organizational needs facing health care workers on a daily basis and the emergence and interpretation of meaning in context.

#### Feeling Part of Organization and Team

Another value that emerged as significant was the feeling of teamwork and togetherness. When team members taught each other, cared about one another, and pulled together in times of need, they felt fulfilled and that they belonged to a community of practice. As one interviewee said, *“*When we are at our best … we’re all clicking together as the teamwork aspect, everybody supporting each other, and that’s how we get through those days”.

Teamwork, which included shared responsibility and goals, commitment to others, compromise/sacrifice, and caring, was considered to be a motivating factor that sustained individuals through difficult day-to-day work and personal crises.

### Value-Challenging WLN Analysis

The value-challenging WLNs were about situations in which employees felt uncomfortable when their values were challenged by others’ behaviors or organizational rules. When confronted with these value-challenging situations, employees reacted in a variety of ways, from experiencing negative emotions (including discomfort and thoughts about quitting work) to behavioral acts meant to change the situation (such as willingness to advocate and fight for patients’ best interests) and even crossing personal boundaries to help meet patients’ needs, by bending organizational rules and regulations at the risk of being fired, in order to stay consistent with their values.

The core values in the 117 value-challenging stories were:

- Treating others with disrespect/respect (respect, acceptance, honesty, and fairness) (45.3%)
- Going above and beyond when values are challenged (flexibility and advocating for others) (14.5%)
- Valuing patients’ well-being (caring and generosity) and willingness to fight for this cause (11.1%)
- Helping and healing (10.3%)
- Doing the right thing (10.3%)
- All others (feeling part of organization and team, expressing passion and emotion) (8.5%)

In an effort to understand further the value-challenging WLNs, we analyzed their characteristics and the general issue(s) constituting the challenge (Table 4).

**Table 4:** Participants and Content of the Value Challenges Work-Life Narratives (WLNs).

**Participants Within WLNs**	**Characteristics and General Issue(s) Constituting the Challenge**	**Number of WLNs**
**Self and patient/family**	Working on self to accept patients’/families’ behaviors	18
	Working on self to accept patients’/families’ values	15
**Self and colleagues**	The way they (i.e. team, supervisor, and physicians) treat me (disrespect, unfairness)	17
	The way they (team and supervisor) treat patients (disrespect)	10
**Self/patient and organization**	Physicians’ violation of patients’ rights for autonomous decisions (dishonesty)	9
	Dealing with strict organizational regulations that reduce the ability to deliver the best care for the patient	18
	Budgetary restrictions/limitations	10

#### The Way We Treat Others and the Way Others Treat Me

Almost half of the value-challenging situations involved respect and focused on the way we treat one another and patients.

#### Challenges of the Narrator (Self) and Patient or Family

In situations concerning self and patient/family, the challenges were mainly about a values conflict between an employee’s own value system and that of the patient (e.g. regarding euthanasia), or a conflict involving unacceptable patient behavior (e.g. a patient acting in anger). Respondents found themselves re-examining their own values and looking for ways to communicate better about values, including the possibility of disagreement. For example:

“A young man in his 20s whose disease was rapidly progressing despite the transplant ended up with an overwhelming infection … on life support on the ventilator. His family made the decision to just keep him comfortable and stop aggressive treatments and the problem was that when they made that decision, they expected [it] to end right then and there and in essence wanting to euthanize him, and that really challenged my core values and that was a big ethical dilemma for me. And we spent the better part of the shift discussing that and explaining and trying to make sure both parties [understood] that.

First and foremost I knew what my own personal values were, and I needed to make sure that it didn’t conflict with the patient and people who need to have an opportunity to express their values as well ... I think the biggest thing was dealing with the family and finding what their fears were, what their worries were, what their concerns were, what their priorities were, and once we got done with that we realized hey let’s just keep him comfortable as long as we know he’s not feeling a thing and we’ll let happen as it needs to happen or as God or nature intends for it to happen.”

In this story, the employee’s self-awareness and an internal and external process of re-examining her own values, as well as considering the family’s values, led to a negotiation process which resulted in a compromise that was congruent with both parties’ values.

#### Others Treating Self, Patients, and Families

Many challenges involving the team, supervisor, or physicians focused on the way co-workers treated the narrator (self) (e.g. when they treated them with little or no respect for their own profession):

“A physician and I did not agree on the approach with a patient at all. And I felt very confident in mine. During that interaction … we were discussing my area of practice, and I just felt not listened to; and I respect the physician and they are the overseer of the patient, but I wasn’t listened to, and I didn’t feel respected. Instead I was being talked down to, and almost yelled at. It wasn’t pleasant. And then comments were made when I wasn’t there, and things were written in the chart that was downplaying my abilities and my role with this patient. And I was really upset. I value communication and I value respect and if you’ve got a problem with me or there is an issue, please come to me and we’ll talk about it. I felt like it affected the working relationship outside of that particular scenario with the patient.”

In this WLN the narrator experienced the cascading effect(s) on her relationship with the physician, other co-workers on the team, and ultimately the patient and the quality of care that was provided. Narratives in this category often described multiple negative effects stemming from a single incident. Clearly, value-challenging experiences such as the one above are of particular concern because they have the potential to affect the quality and delivery of patient care.

Some value-challenging WLNs also focused on the disrespectful treatment of patients’ or families’ life-styles or sexual orientations, as illustrated below:

“There was a transgendered individual on our unit. Two staff members weren’t tolerant of the situation, and, in return, I was pretty adamant about how I felt that they became very judgmental. I guess I was trying to be supportive of this person … The other staff member made a comment about Jesus and fags, and I was just like, ‘Oh, we don’t use that word’. And she thought it was very acceptable to say that about a patient … [and to] refuse to address that patient as whatever gender they had decided that they were.”

The way in which the narrator’s colleagues failed to recognize the value of respectful treatment for all patients, irrespective of their station in life or sexual orientation, created a values conflict for which there does not seem to be solution. Rather, there is an unstated impasse which could have a negative effect on the team’s ability to care for the next transgendered individual or any patient who elicits conflicting values.

Similarly described were situations in which physicians ignored patients’ rights:

“… we have patients that are quite elderly that have a cancer diagnosis and the doctors are very aggressive in their treatment in providing chemotherapy and that they wait way too long before they get hospice involved so the patients can actually have that good death and be at home like they want to be. I am challenged with that almost every day in trying to be a patient advocate … even recently a patient said, ‘Will you please be there when the doctor makes rounds tomorrow so that he doesn’t talk me out of this, again. Will you please be there? I just want to go home …’ … it’s not about what I want, it’s about what the patient wants, and so I try to make sure the patient has all the information and that the doctor knows what the patient wants, and the patient is clear about that and we’re all on the same page with it.”

This is another example of mindfulness or self-awareness achieved by creating a communication process for re-examining one’s own and others’ values. When the patient felt that the physician was trying to impose his/her own values, s/he asked the nurse to assume a supportive role as a mediator, bringing the value conflict out in the open and then placing the patient’s values first. When faced with these types of situations, many narrators took it upon themselves to advocate for patients’ rights. This story also illustrates how a value-challenging situation can reach a resolution where everyone feels comfortable with the outcome. Resolutions were reached only in one-sixth of all the challenging stories.

#### Going Above and Beyond

Most of the WLNs that were categorized as ‘going above and beyond’ were related to self/patient and the organization. These stories included advocating on patients’ behalf, fighting for preserving good-quality care, and being creative in finding ways to help in spite of organizational rules and regulations. As opposed to value-affirming WLNs of going above and beyond that were creative and fun, the value-challenging WLNs included violating hospital regulations to assist a patient, as illustrated in the following example:

“I think sometime you have to kind of walk the line versus hospital politics, like possibly a patient leaving the hospital knowing they don’t have resources, maybe you can help them out, there are things that you have to do that is not protocol, but it’s in the best interest of the patient or person. So you do it. One example is that you are never supposed to go to a person’s house to do something. We had an IV line still in him that we forgot to take out and the family said they couldn’t bring their loved one back to the hospital. So I went to that resident and removed it …”

The employee stressed the fact that she broke the hospital regulations when it meant acting in the patient’s and family members’ best interest.

#### Value-Challenges with the Organization

Value-challenging WLNs regarding difficulties with the organization focused mainly on strict regulations and budgetary issues that contradicted or negatively affected the teller’s perception of high-quality patient care, as illustrated in the following WLN:

“A challenge for me is when we get patients and you can’t adequately take care of them due to staffing situations. In the last few years, the main focal point that is projected is budgetary. Still the desire to give adequate, certainly a high standard and high level, which I think staff does because of this commitment, but the commitment to reinforce that in a meaningful way seems to have diminished from management, because they have to focus solely on budget issues. It’s hard to treat people like factory assembly work; get in and get out and go on to the next task.”

This narrator experienced conflicting forces: one committed to the patient, the person, and adequate care, the other committed to budgetary issues and saving money. The value challenge emphasized here was facing human beings who work for/with human beings, rather than machinery.

### Resolutions

Almost two-thirds (60%) of the value-challenging situations were not handled in a constructive way, and no resolution was indicated. We identified resolution situations in stories where a satisfying conclusion or understanding was achieved. The following is a list of characteristics in stories in which no resolution was achieved:

- Most self and supervisor (75%) value challenges were unresolved.
- Two-thirds (65%) of value challenges between self and team were unresolved; another 26% were only partially resolved.
- Two-thirds (63%) of the self and patient/family value challenges were unresolved.
- More than half (59%) of the self/patient/team and organization values challenges were unresolved.

Of the remaining value-challenging situations a small number were partially resolved through some conversation about the conflict. However, the narrators of these stories were still left with uncomfortable feelings or a fear of recurrence of the challenge.

Narratives in which employees felt that a resolution was achieved included situations in which the rules or regulations had been bent or the boundaries stretched to help a patient in what the narrator believed was the patient’s best interest. These were referred to as “win–win” situations. Other cases were resolved by debriefing, discussing and acknowledging the issue, or by creating new rules and regulations to address it.

### Comparison between Value-Affirming and Value-Challenging WLNs

A comparative matrix (Table 5) illustrates how most value-affirming WLNs were focused on the self/team and the patient/families as compared with the value-challenging WLNs that were mainly about the self/patient and the organization, supervisor, or physicians.

**Table 5:** Value-challenging and value-affirming characteristic matrix (percentage).

	**Type of Value Story**	**Patient (%)**	**Family (%)**	**Team (%)**	**Supervisor (%)**	**Physician (%)**	**Organization (%)**
**Self**	Challenging	12.8	10.3	16.2	9.4	8.7	23.0
	Affirming	38.5	9.0	18.4	0.6	0.6	9.0
**Patient**	Challenging			6.8			9.4
	Affirming			17.8			1.2
**Family**	Challenging						
	Affirming				2.5		0.6
**Team**	Challenging						3.4
	Affirming						1.8

In addition, a comparative matrix of the value-affirming and value-challenging core values (Table 6) shows that the majority of value-challenging WLNs centered on respect, whereas the bulk of value-affirming stories focused on going above and beyond, valuing patients’ well-being, helping and healing, and feeling a part of the organization and team.

**Table 6:** Value-challenging and value-affirming core value matrix in percentages

**Value**	**Affirming, *n* (%)**	**Challenging, *n* (%)**
**Going above and beyond**	41 (24.2)	17 (14.5)
**Valuing patients’ well-being**	35 (20.7)	13 (11.1)
**Helping and healing**	34 (20.1)	12 (10.3)
**Feeling part of organization and team**	29 (17.2)	8 (6.8)
**Gratitude and appreciation**	11 (6.5)	0 (0)
**Believing in a higher power**	9 (5.3)	0 (0)
**Expressing passion and emotion**	4 (2.4)	2 (1.7)
**Doing the right thing**	2 (1.2)	12 (10.3)
**Treating others with disrespect/respect**	2 (1.2)	53 (45.3)
**Growing and developing**	2 (1.2)	0 (0)
**Total (*n*, %)**	**169 (100)**	**117 (100)**

## DISCUSSION

Our findings demonstrate the important role of values in day-to-day work and the role of personal narratives in illuminating the complex and often competing demands of the workplace. Personal values guide decision-making, influence behavior in caring situations, and produce positive effects when individual and institutional values are aligned. By the same token, when personal values are challenged, employees might experience conflicting emotions, with a cascade of negative effects. Under challenging conditions, employees were willing to fight for their personal values, even at the risk of losing their jobs. As earlier findings indicate and our findings illustrate: “Individuals take risks, overcome barriers, relinquish their own comfort and security, and generate extraordinary effort because of their values”.^22^

The majority of value-affirming WLNs focused on times and experiences associated with providing direct care to patients and families. We note that the four values found in this category, going above and beyond, valuing patients’ well-being, helping and healing, and being part of a team and organization, occurred in roughly equal proportions. We suspect that a close tie exists between personal and professional values when it comes to providing high-quality care and that high-performing employees believe that they are fulfilling their roles by delivering care that is consistent with their own and the organization’s values. The idea of placing patients’ best interest above all other values made employees feel at their best. This finding is consistent with recent findings indicating that top-performing organizations were those whose personnel have a shared value of top-quality care for patients.^23^ Working according to this value leads to an on-going improvement process.

By contrast, the majority of value-challenging stories were about difficulties that employees encountered with their co-workers, including physicians, and rules and regulations of the organization. These stories focused on disrespectful behaviors toward the self and others that elicited negative feelings and behaviors. This is a worrisome finding, due to recent findings that indicate that respect for patients’ feelings is known to be a significant attribute of measurement of quality of care.^24^ Other studies show that disrespect is associated with low-performing organizations that leads to isolation and interdependencies^23^ and negative emotional responses, such as hurt feelings or mistrust.^25^

An additional concern is that most of these negative experiences remained unresolved. The lack of resolution in so many WLNs could have significant implications for employees, patients, and the organization as a whole. It is not far-fetched to imagine that the next time a transgendered individual comes to the ward in which a challenging story was told, their care might be compromised by underlying unresolved issues among staff members. Other unresolved value conflicts could result in negative feelings toward the organization, a supervisor, or team members, leading to feelings of dissatisfaction, staff turn-over, diminished quality of care, and lower performance level.^23^ Unresolved incidents and poor communication concerning them diminishes the ability to learn, change, and develop in the hospital environment.

We began with the observation that current methods for understanding workplace satisfaction might miss some of the lived experience and contextual meaning experienced by employees in their daily work. We deliberately focused on high-performing employees to understand what they report when they are at their best and when they are challenged. We found that even the best employees have difficulty resolving challenging situations in the workplace. This leads us to suggest that education in polarity management and ways of holding difficult conversations might be used to challenge and change the prevailing culture of silence in the face of challenge.

Two subcategories of values that were salient were creativity and flexibility in day-to-day work. Value-affirming stories focused on being able to create a better environment of care by bending the rules. Also, value-challenging stories were often handled by bending the rules. The ability to bend the rules made employees feel more comfortable and more autonomous in their jobs. Kelly^26^ warns against the trend of hospitals to give nurses less and less room for a personalized approach to clinical practice. The solution suggested for this problem was the acknowledgment of nursing values that can be implemented in health care. Our findings support this suggestion and demonstrate, paradoxically, that when employees “follow” their values and act according to them, they are actually implementing the organization’s values, while at the same time violating formal organizational rules and regulations. As has been demonstrated in other studies, “stories reveal the gap between the formal codified space of an organization (roles, job descriptions, and lines of accountability) and informal un-codified space (relationships, feelings, ‘unwritten rules’, and subcultures)”.^27^^(p. 444)^ The informal code among those interviewed was to place the patients’ best interests above total compliance with the organization’s rules and regulations. Our findings suggest the importance of encouraging flexibility and creativity in providing the best quality of care possible. Rigid conformity to the rules may be viewed as a barrier to high-quality care, while flexibility (with an appropriate degree of accountability) to bend the rules is appreciated by employees, patients, and families.

The present study has several limitations that should be noted. First, by focusing on high-performing employees, we cannot and did not attempt to be representative of the entire employee population. Future studies of these populations should be performed. Furthermore, this study was carried out in only one health science center, with a fairly small sample, using a snowballing sampling design. Future research in this area could include comparative studies of larger populations stratified by professional roles (including physicians), age, and value orientations. Additional studies might also link our type of narrative approach with organization-wide staff satisfaction and culture surveys in order to create a more holographic image of what gives life and meaning to the organization from 30,000 feet to ground level, and allow capturing the complexity in day-to-day work in a health care organization — something that is unlikely to be captured in a workplace satisfaction questionnaire.

The strength of the study is in the use of the workplace narrative method, which proved to be an important vehicle for identifying underlying value structures that can be used to celebrate successes, find “hot spots”, and point the way to better alignment of organizational goals through personal experience. Our study showed how the use of appreciative and challenging qualitative narrative data collection and analysis can provide an opportunity to identify what really matters to health care professionals within the organization as well as obstacles to change,^11^ such as inflexibility in hospital regulations, or lack of resolution opportunities and tools. As Taylor and Keighron^28^ wrote from their experience, “listening, honoring, and retelling our stories reaffirms the lessons we have learned in our journey” (p. 246), thus reminding us of the potential hidden in using these stories to begin this process. WLNs provide insight into the complexity of health care and the intensely personal ways in which employees derive meaning and predicate their actions in context. Changing the nature of the conversations and stories that people tell in an organization is one means of transforming our understanding of health care as a form of bureaucracy, complete with formalized rules and regulations, to a human endeavor wherein persons in distress seek the help of qualified professionals, one story at a time.

## Abbreviations:

WLNs: work-life narratives.
